# Supplementary material for: Aurora Kinase A inhibition enhances DNA damage and tumor cell death with 131I-MIBG therapy in high-risk neuroblastoma
Source: EJNMMI Res. 2024 Jun 13;14:54. doi: 10.1186/s13550-024-01112-7 (PMC11176152; doi:10.1186/s13550-024-01112-7)
Supplement: Supplementary file 1 — Additional file 1. Supplemental Data. [file 13550_2024_1112_MOESM1_ESM.docx]

Supplemental Data

Aurora Kinase A inhibition enhances DNA damage and tumor cell death with ^131^I-MIBG therapy in high-risk neuroblastoma

Prerna Kumar, MD^+1,2^; Jessica Koach, PhD^+2^; Erin Nekritz^2^; Sucheta Mukherjee, PhD^2^; Benjamin S. Braun, MD, PhD^2,7^; Steven G. DuBois, MD, MS^3^; Nicole Nasholm^2^; Daphne Haas-Kogan, MD^4^; Katherine K. Matthay, MD^2,7^; William A. Weiss MD, PhD^2,5,7^; Clay Gustafson, MD, PhD*^2,7^, Youngho Seo, PhD*^6,7^;

+ Co-first authors

* Co-senior authors

Materials and Methods

*Cell culture*

SK-N-BE(2) neuroblastoma tumor cell line was obtained from the Childhood Cancer Repository, Children’s Oncology Group resource laboratory. Kelly cells were purchased from Sigma-Aldrich. NB1691-Luc cell line was received from Peter Houghton’s lab at University of Texas San Antonio. We transduced these cells to over express hNET as described above to enhance MIBG uptake^26^. All neuroblastoma cells were grown in DMEM media (Gibco, Thermo Fisher) with 10% FBS, except for Kelly cells, which were grown in RPMI media (Gibco, Thermo Fisher) with 10% FBS. Cells were maintained at 37^o^C in humid air with 5% CO_2_.

*Cell viability assay*

Neuroblastoma cells, 1x10^4^ cells, were pre-seeded into 96-well white plates for 24 hours prior to alisertib (S1133, Selleck chemicals) and LY3295668 (S8782, Selleck chemicals) treatment for 4 hours followed by external beam radiation (4 Gy). Cell viability was measured 72 hours post treatment using CellTiter-Glo assay (Promega) according to the manufacturer’s protocol. Luminescence was read on the Synergy Neo2 microplate reader (BioTek).

*Immunofluorescence*

Cells were pre-seeded (2x10^5^ cells per well) on glass coverslips, in 6-well plates. 24 hours post seeding, cells were treated with various concentrations of alisertib (S1133, Selleck chemicals) for either 24 hours (for G2/M cell cycle arrest) or for 4 hours followed by external beam radiation (4 Gy) (for DNA damage analysis). Nocodazole (0.5ug/ml, 16 hours) was used as positive control to arrest cells in G2/M. Cells were fixed with 4% paraformaldehyde (Thermo Fisher) for 10 minutes then permeabilized in 0.1% Triton X-100 for 5 minutes. Normal goat serum (5% in 0.1% Tween) was used to block the cells for 30 minutes before overnight incubation with primary antibodies; α-tubulin (236-10501, Thermo Fisher), pAURKA (C39D8, Cell Signaling Technology), γH2AX (Ser139 (20E3), Cell Signaling Technology), 53BP1 (NB100-304, Novus). Cells were incubated with secondary antibody; AlexaFluor 488-rabbit, or AlexaFlour 568-mouse (Molecular Probes) for 2 hours then mounted onto glass slides with mounting medium containing Dapi (H-1200, Vector Laboratories). Slides were imaged on the Leica DMi8 fluorescence microscope at 40x and 60x magnification. Quantification of arrested cells and the number of DNA damage foci markers were performed using Fiji Image J software, where more than 100 cells were analyzed for each treatment and the results were expressed as percentage of total cells analyzed or average number of foci per nuclei.

*Western blotting*

Cells were lysed with RIPA lysis buffer (Sigma-Aldrich) containing protease inhibitor (Sigma-Aldrich) and phosphatase inhibitor cocktail (Roche). Western blots were performed using standard protocol. Briefly, protein lysates (30µg) were resolved on 4-20% SDS-polyacrylamide Criterion midi gels (Bio-Rad) and transferred to a nitrocellulose membrane using the Trans-Blot Turbo (Bio-Rad). Membranes were blocked with 10% BSA for 1 hour. All antibodies were purchased from Cell Signaling Technology including Chk2 (D9C9), p-Chk2, pH2AX (Ser139), Histone H3 (3H1), p-Histone H3 (S10), Aurora A, p-Aurora A/B/C (2914), cleaved PARP (D1214), cleaved caspase-3 (D175), except GAPDH (Millipore) and MYCN (B8.4.B, Santa Cruz Biotechnology). Secondary antibodies (anti-rabbit-HRP and anti-mouse-HRP) were purchased from Cell Signaling Technology. Protein detection was performed using Clarity Western ECL Substrate (Bio-Rad) and visualized on the ChemiDoc imaging system (Bio-Rad).

*Flow cytometry*

Kelly, SK-N-BE(2) (2x10^5^ cells per well), and NB1691-LUC (4x10^5^ cells per well) cells were pre-seeded in 6-well plates for 24 hours prior to alisertib (S1133, Selleck chemicals) treatment.

*Cell cycle arrest analysis*

Cells were treated with alisertib (100 nM) for 4 and 24 hours then harvested and washed with PBS followed by one wash with 1% BSA in PBS. Cells were fixed with 4% paraformaldehyde (Thermo Fisher) in PBS for 15 min at room temperature covered from light and permeabilized with methanol for 15 min at 4°C covered from light. Cells were stained with 0.5 μg/mL DAPI (Life Technologies, D21490) and analyzed with the BD LSR II flow cytometer (BD Biosciences). Analysis of flow cytometer data was performed using Cytobank software (http://www.cytobank.org).

*Cell apoptosis analysis*

Cells were treated with alisertib for 4 hours followed by external beam radiation (4 Gy). Cells were harvested 48 and 72 hours post treatment and washed in PBS then stained with APC-Annexin V and propidium iodide (PI) apoptosis detection kit (BioLegend). Flow cytometry was performed on the BD Accuri C6 plus flow cytometer (BD Biosciences) and data was analyzed using FlowJo software.

*Immunohistochemistry*

Xenograft neuroblastoma tumors, treated with alisertib and MIBG, were excised from mice and paraffin fixed for Hematoxylin and Eosin staining and analysis of cleaved caspase-3 expression using standard immunohistochemistry protocols. Briefly, paraffin embedded tissues were deparaffinized by heating samples at 65^o^C for 1 hour followed by rehydration with xylene and ethanol. Antigen retrieval was performed by incubating slides in 3% hydrogen peroxide for 10 minutes followed by blocking for 30-60 minutes in 10% NGS (diluted in 0.1% Tween TBS). Cleaved caspase-3 antibody (1:100 dilution, D175, Cell Signaling Technology) was applied to tissues and incubated in a humidity chamber overnight. Slides were washed with PBS then incubated for 60 minutes with SignalStain Boost HRP rabbit reagent (Cell Signaling Technology). Peroxidase Substrate solution was used for measuring protein expression. Slides were counter stained with hematoxylin and visualized on the Leica DMi8 microscope.

*In vitro radiation*

Radiation was administered via a Cesium-137 irradiator. Recent half-life studies approximated that 1 Gy would be delivered in 0.35 minutes. Therefore, cells were irradiated for 1.4 minutes to receive 4 Gy. Synergy calculations can be completed using the Chau-Talalay formulas with three-dimensional isobolograms demonstrating that two compounds are either additive, synergistic, or antagonistic. These calculations, however, require a known EC_50_ for both compounds in order to calculate the threshold (slope on the graph) by which these different responses are measured. Because external beam radiation does not classically have a true EC_50_, isobologram calculations could not be done with *in vitro* combination treatment with external beam radiation and targeted small molecule compounds. Therefore, to serve as a surrogate for synergy, dose response curves of drug were analyzed with and without external beam radiation to observe for leftward shifts of the EC_50_ , since a decreased EC_50_ for a given drug suggests that less drug is needed to achieve the same effect when given in combination with another agent.

*In vivo studies*

NOD SCID gamma mice (Jackson Laboratory) were implanted with 2x10^6^ NB1691-LUC/NET neuroblastoma cells in growth media with 50% Geltrex (Invitrogen) subcutaneously to enable accurate measurement^29^. Tumor bearing mice were treated with alisertib (20mg/ml) 20mg/kg for 7 days or saline control, via intraperitoneal (IP) injection. IP injection, which has been used previously^31,32^, was used to minimize the radiation exposure of the handler while dosing. The combination and MIBG cohorts received 37 MBq (1 mCi) of ^131^I-MIBG 24 hours after the first dose of alisertib or alisertib carrier for the MIBG alone arm). Tumor size was assessed twice weekly. Tumor growth was monitored for up to 25 days for all three groups. Mice were euthanized once maximum tumor length reached 2.0 cm in long axis. After 26 days, mice were harvested, tumors were fixed, and ^131^I-MIBG was allowed to decay. Tumor growth was analyzed by a linear mixed effects model, similar to that described by Akutagawa et al^33^. Tumor volume, as calculated from caliper measurements, was transformed by square root to correct for heteroscedasticity and normalize residuals. This was achieved with the exception of a single outlier. Logarithm transformation was rejected due to the presence of data points with tumor volume of 0 when tumors were undetectable. Fixed effects included assigned treatment and time, which was included as first and second-degree orthogonal time-dependent polynomials. Random effects were included for individual mice. Confidence intervals were estimated by the bootstrap method at the 95% level. At least one time-dependent coefficient for the cohort treated with MIBG and alisertib was different from coefficients for all other groups, based on these 95% confidence intervals. All experiments on live vertebrates were performed in accordance with relevant institutional and national guidelines and approved by the UCSF Animal Care and Use Committee (IACUC).

Data

*Gels/blots*

Figure 4 Treatment with LY3295668 and radiation in neuroblastoma cell lines induces cell death


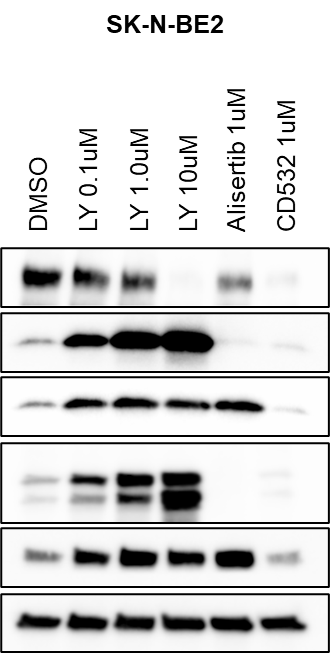

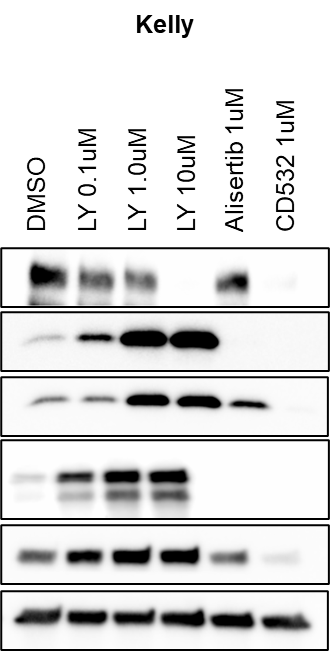

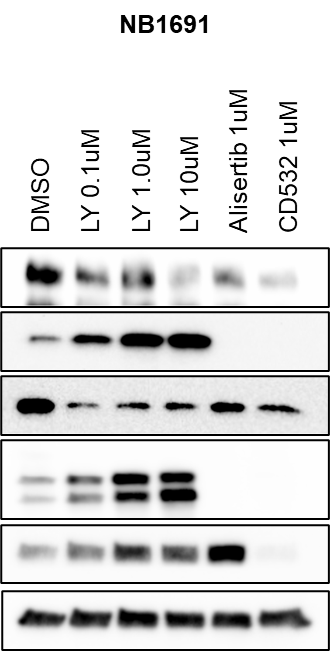


B

N-myc

pH3

H3

GAPDH

pAURK B & C

AURK A

**Figure 4 Treatment with LY3295668 and radiation neuroblastoma cell lines induces cell death**

A. Dose response of LY3295668 with and without radiation across three MYCN amplified cell lines pre-treated with LY3295668 followed by radiation showed a lower IC50 concentration. Data for IC50 concentrations were normalized to account for the effect from radiation alone. B. Immunoblots of cells treated with LY3295668 show that treatment increased N-myc and AURKA degradation in a dose-dependent manner.

**
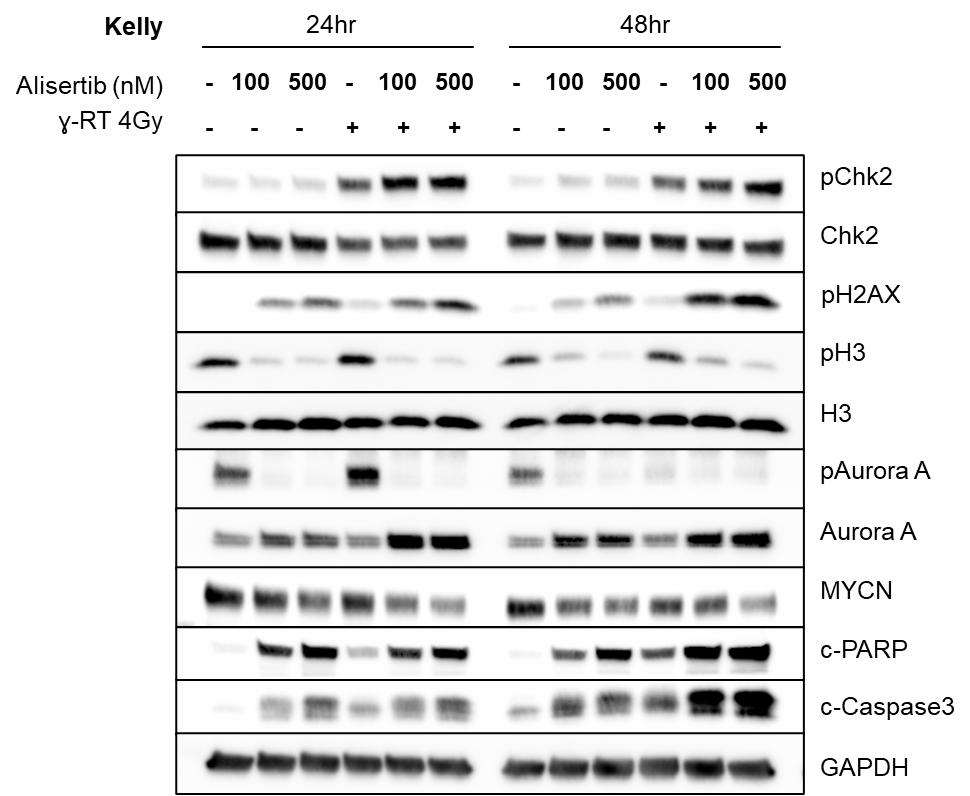

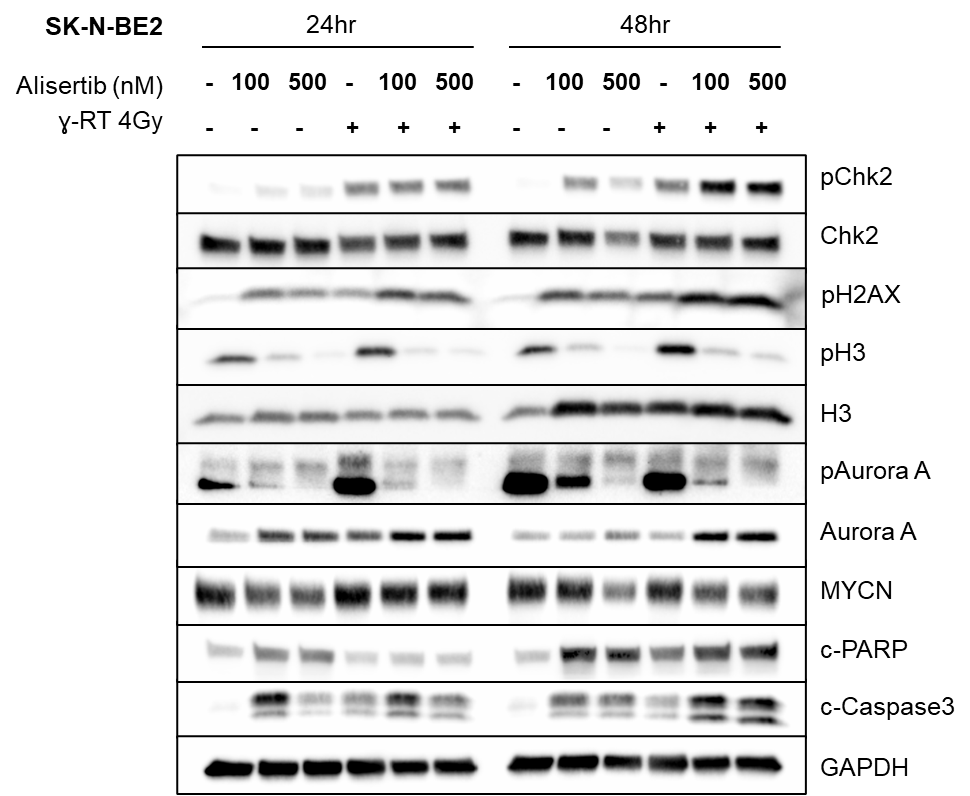

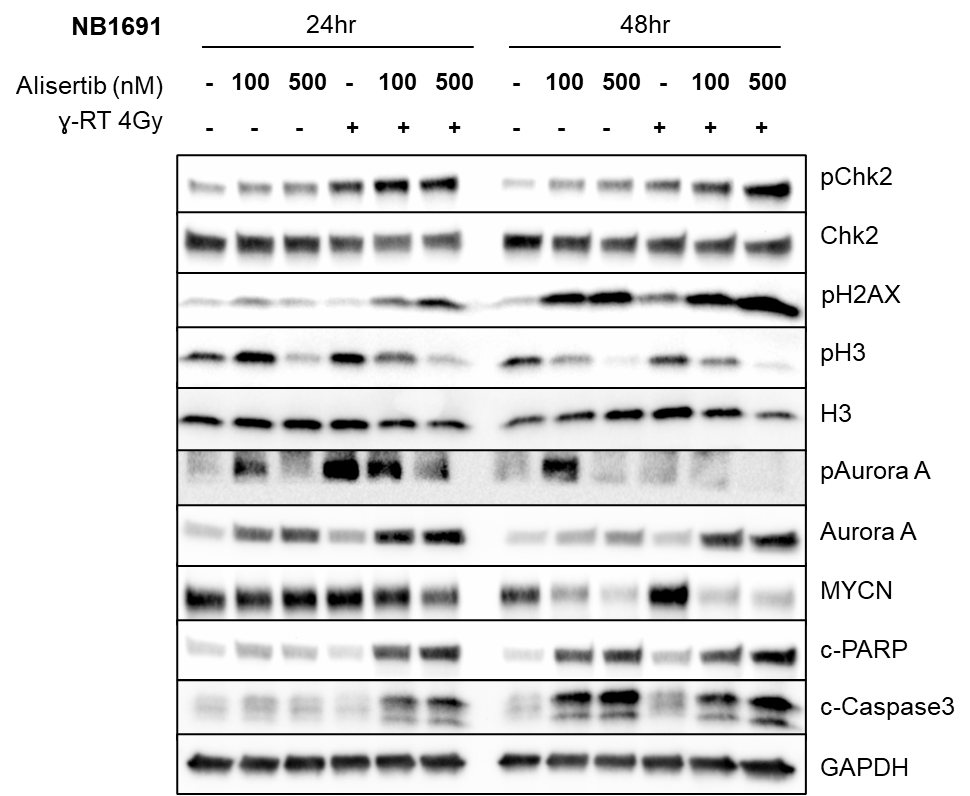
**

Figure 6 Alisertib and radiation therapy potently induces apoptosis, downregulates MYCN protein, increases DNA damage, and enhances/prolongs Chk2 activation

**Figure 6 Alisertib and radiation therapy potently induces apoptosis, downregulates MYCN protein, increases DNA damage, and enhances/prolongs Chk2 activation**

Immunoblots of cells treated with alisertib and radiation show that treatment increases and prolongs pChk2 and pH2AX expression, decreases pH3 and pAURKA, and increases cleaved PARP and cleaved caspase-3 expression.
